# Changes in the clinico-functional characteristics of stroke patients in the acute phase during the COVID-19 pandemic

**DOI:** 10.31744/einstein_journal/2023AO0226

**Published:** 2023-06-06

**Authors:** Jordana de Paula Magalhães, Iza de Faria-Fortini, Zaqueline Fernandes Guerra, Nathália Aparecida Gravito Rodrigues, Romeu Vale Sant’Anna, Christina Danielli Coelho de Morais Faria

**Affiliations:** 1 Universidade Federal de Minas Gerais Belo Horizonte MG Brazil Universidade Federal de Minas Gerais, Belo Horizonte, MG, Brazil.; 2 Hospital Risoleta Tolentino Neves Belo Horizonte MG Brazil Hospital Risoleta Tolentino Neves, Belo Horizonte, MG, Brazil.

**Keywords:** Stroke, SARS-CoV-2, COVID-19, Coronavirus infections, Pandemics, Public health, Health services

## Abstract

**Objective:**

To compare the sociodemographic and clinico-functional characteristics of patients admitted to a stroke unit immediately before and during two different COVID-19 pandemic phases.

**Methods:**

This exploratory study was conducted in the stroke unit of a public hospital in Brazil. Patients consecutively admitted to a stroke unit for 18 months with primary stroke aged ≥20 years were included and divided into three groups: G1: Pre-pandemic; G2: Early pandemic; and G3: Late pandemic. The sociodemographic and clinico-functional characteristics of the groups were compared (α=0.05).

**Results:**

The study included 383 individuals (G1=124; G2=151; G3=108). The number of risk factors (higher in G2; p≤0.001), smoking (more common in G2; p≤0.01), type of stroke (ischemic more common in G3; p=0.002), stroke severity (more severe in G2; p=0.02), and level of disability (more severe in G2: p≤0.01) were significantly different among the groups.

**Conclusion:**

A greater number of serious events and risk factors including smoking and higher level of disability was observed in patients in the beginning of the pandemic than in the late phases. Only the occurrence of ischemic stroke increased in the late phase. Therefore, these individuals may have an increased need for rehabilitation services monitoring and care during their lifespan. Additionally, these results indicate that health promotion and prevention services should be strengthened for future health emergencies.

## Highlights

■ The clinico-functional characteristics of post-stroke patients changed in the pandemic.

■ The sociodemographic characteristics of post-stroke patients did not change in the pandemic.

■ Post-stroke patients had a greater stroke severity and disability at the beginning of the pandemic.

■ The occurrence of ischemic stroke increased with the advancement of the pandemic.

**Figure:**
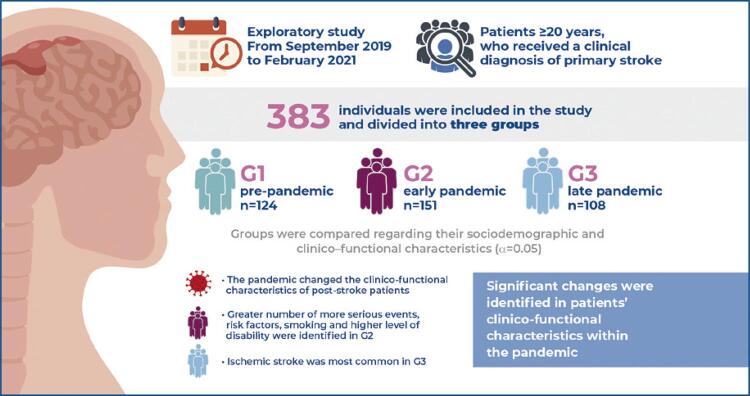

## INTRODUCTION

The coronavirus disease 2019 (COVID-19) pandemic generated unprecedented demand for health services in many several countries.^(1)^ Even after two years since the start of the pandemic, healthcare for COVID-19 patients and those with other health conditions, such as stroke, remains a challenge.^(2,3)^ Studies conducted by professionals involved in stroke units have shown that even in high-income countries, the pandemic has hindered patient care, including handling neurovascular damage and rehabilitation process.^(2,4,5)^ Admissions for stroke have markedly declined worldwide during the COVID-19 pandemic.^(4,5)^ Moreover, an increase in the number of severe cases was observed.^(6)^This decrease in hospital admissions could reflect a reluctance to call emergency services. Additionally, a delay in admission for acute care due to stroke negatively impacts the survivors’ health.^(4,5,7)^

Among middle-income countries, Brazil has one of the highest numbers of cases and deaths due to COVID-19.^(8)^ Brazil accounted for 24 million infected people and 623 thousand deaths due to COVID-19 in early 2022 which inevitably impacted the country’s health system, especially emergency units. Previous studies conducted in Brazil showed a decrease in cases of transient ischemic attack and acute ischemic stroke at the beginning of the pandemic.^(9,10)^ These studies explicitly focused on transient ischemic, acute ischemic, mild, and moderate strokes and considered distinct, non-consecutive phases of the pandemic.^(9,10)^ In addition, these studies were carried out in two medium-sized cities with high levels of human development in relation to national numbers, which do not represent the complete sociodemographic characteristics of Brazil.^(9,10)^

In Brazil, stroke is one of the main causes of hospitalization in the public health system and one of the leading causes of disability.^(11)^ In addition to the large expenses associated with the care of acute stroke patients, individuals with stroke commonly need continuous care after hospital discharge and contribute to a majority of the disease burden in Brazil.^(11,12)^ More than 60% of stroke patients receive rehabilitation services after discharge. As recommended by international guidelines^(13,14)^ and the Ministry of Health of Brazil,^(11,12)^ individuals with stroke should be integrally and continuously monitored by rehabilitation services after discharge.^(11-14)^ Therefore, we analyzed the clinico-functional characteristics of individuals affected by stroke in the different phases of the pandemic to understand its impact on the acute phase of stroke and identify the healthcare needs of stroke patients. Moreover, this study can be useful in planning healthcare and managing public policies related to chronic stages of stroke, even after the end of the pandemic.

## OBJECTIVE

To compare the sociodemographic and clinico-functional characteristics of patients admitted to a stroke unit of a public hospital from an important metropolis in Brazil immediately before and in two different phases of the COVID-19 pandemic.

## METHODS

### Study design and settings

This exploratory study was conducted in Belo Horizonte, one of the largest metropolises in Brazil. This study was approved by the institutional research ethical committees of the *Universidade Federal de Minas Gerais* (UFMG) and the *Unidade de Acidente Vascular Cerebral do Hospital Risoleta Tolentino Neves* (UAVC/HRTN), hospital where the study was carried out (CAAE: 84263818.8.0000.5149, # 2.568.736). All the participants or their proxies provided written informed consent.

### Participants and procedures

The participants were screened and recruited after admission to a stroke unit of a public emergency hospital. To obtain a representative sample, the consecutive patients were recruited^(15)^ between September 26^th^, 2019, to February 26^th^, 2021, which encompassed the phases before and after the beginning of the pandemic.^(16)^ The first reported case of severe acute respiratory syndrome coronavirus 2 (SARS-CoV-2) infection in Brazil was February 26^th^, 2020.^(16)^ Individuals were divided into three groups according to the date of hospital admission: Group 1, G1: Pre-pandemic (patients admitted between September 26, 2019, and February 25, 2020); Group 2, G2: Early pandemic (patients admitted between February 26^th^ and September 25^th^, 2020); and Group 3, G3: Late pandemic: patients admitted between September 26^th^, 2020, and February 25^th^, 2021.

Patients ≥20 years of age who received a clinical diagnosis of primary stroke, as confirmed by neuroimaging examination, were invited to participate. Those who had previous incapacity, defined by a Barthel Index score ≤17,^(17)^or already had cognitive impairment, defined by a Heteroanamnesis List Cognition score >1 derived from the Mini Mental State Examination,^(17)^were excluded.

### Data sources and measurements

Sociodemographic and clinico-functional characteristics were obtained from the patients’ medical records at their hospital discharge. Sociodemographic data included sex, age, and socioeconomic status.^(18)^ Clinico-functional data included length of hospitalization, number and type of risk factors, number of medications, stroke type, thrombolytic therapy, stroke severity (National Institute of Health Stroke Scale), and level of disability (Modified Rankin Scale).^(19)^ All data were collected by Two examiners were trained for two weeks (12 hours of training) on using the procedures of this study for data collection. The training was performed by the main researcher of this study, as per previous procedures and recommendations.^(17-19)^ These examiners and principal investigator had more than five years of clinical and/or research experience in the area of stroke rehabilitation and worked in the stroke unit where the study was developed. Finally, data collection began only after these two examiners showed adequate consistency in the procedures and recommendations.^(17-19)^

### Sample size and statistical analysis

All consecutive patients admitted to the stroke unit between September 26^th^, 2019 to February 26^th^, 2021^(15)^ who agreed to participate and met all eligibility criteria were included. Sample sizes for between-group comparisons were determined using G Power software, version 3.1.9.4 (Franz Faul, Kiel, Germany) considering α=5%, β=80% and a medium effect size (d=0.5). In total, each group had at least 66 subjects.

The distribution of quantitative data was verified using the Kolmogorov-Smirnov test. Descriptive statistical analysis was performed considering the mean and standard deviation for numerical variables with normal distribution, median and interquartile difference for numerical variables with non-normal distribution, and absolute and relative frequencies for categorical variables. Inferential statistics were used for between-group comparisons (χ^2^ or analysis of variance [ANOVA] tests, followed by post hoc tests). All statistical analyses were conducted using SPSS for Windows (version 17.0, SPSS Inc., Chicago, Illinois, United States) (α=5%). Statistical significance was set at p<0.05.

## RESULTS

**Figure 1:**
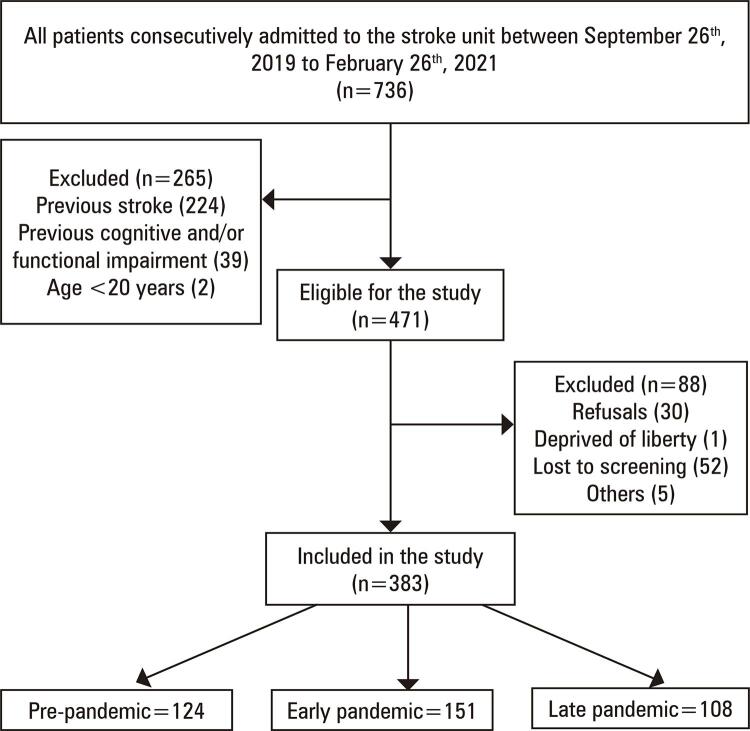
Flowchart of participant selection in the study

In this study, 736 patients were admitted to the stroke unit, of whom 383 were finally included: G1=124, G2=151, and G3=108 (Figure 1). The sociodemographic and clinico-functional characteristics of the participants are shown in table 1.

**Table 1:** Sociodemographic and clinico-functional characteristics of the participants

Variables	Pre-pandemic (n=124)	Early pandemic (n=151)	Late pandemic (n=108)	p value
Sex n (%), male	64 (51.6)	70 (53.6)	53 (50.9)	0.89^‡^
Type of stroke n (%), ischemic	109 (87.9)	128 (84.8)	103 (95.4)	0.02*^‡^
Thrombolytic therapy n (%), no	107 (89.9)	121 (84)	81 (83.5)	0.29^‡^
Reported risk factors n (%)				
Alcoholism	31 (25.2)	41 (27.2)	23 (21.3)	0.55^‡^
Chronic obstructive pulmonary disease	2 (1.6)	6 (4)	2 (1.9)	0.40^‡^
*Diabetes mellitus*	30 (24.2)	40 (26.5)	26 (24.1)	0.87^‡^
Heart disease	13 (10.5)	15 (9.9)	9 (8.3)	0.84^‡^
Hyperlipidemia	4 (3.2)	1 (0.7)	1 (0.9)	0.19^‡^
Hypertension	82 (66.1)	102 (67.5)	64 (59.3)	0.35^‡^
Obesity	8 (6.5)	4 (2.6)	5 (4.6)	0.31^‡^
Sedentary lifestyle	95 (78.5)	119 (78.8)	87 (80.6)	0.91^‡^
Smoking	36 (29.3)	89 (58.9)	26 (24.1)	<0.01*^‡^
Stroke severity, n (%)^† §^				0.02*≠
Mild	62 (50)	60 (39.7)	58 (53.7)	
Moderate	49 (39.5)	64 (42.4)	40 (37)	
Severe	12 (9.6)	27 (17.9)	10 (9.3)	
Level of disability, n (%)^#^				<0.01*≠
Asymptomatic	19 (15.3)	2 (1.3)	7 (6.5)	
Symptoms without disability	20 (16.1)	14 (9.3)	29 (26.9)	
Slight disability	25 (20.1)	23 (15.2)	15 (13.8)	
Moderate disability	12 (9.7)	16 (10.6)	15 (13.8)	
Moderately severe disability	35 (28.2)	39 (25.8)	26 (24.1)	
Severe disability	13 (10.6)	57 (37.8)	16 (14.9)	
Socioeconomic status, n (%)^£^				0.11≠
A	1 (0.8)	0 (0)	0 (0)	
B	23 (18.5)	27 (17.8)	11 (10.2)	
C	61 (49.2)	97 (64.3)	68 (63)	
D	31 (25)	23 (15.3)	29 (26.8)	
E	2 (1.6)	3 (2)	0 (0)	
Length of hospitalization (days), median (IQR)	10 (8.75)	10 (12)	10 (12)	0.35≠
Age (years), median (IQR)	64 (17)	62 (21)	62 (16.7)	0.97≠
Number of risk factors, median (IQR)	2 (1)	3 (1)	2 (2)	<0.01*≠
Number of medications, median (IQR)	2 (3)	2 (3.75)	2 (4)	0.51≠

^‡^ χ^2; ≠^ANOVA; * p<0.05; ^†^ National Institutes of Health Stroke Scale; ^#^ Modified Rankin Scale; ^§^ Not reported n (%): pre-pandemic = 1 (0.9); ^£^ Not reported n (%): pre-pandemic = 6 (4.9), early pandemic = 1 (0.6).

IQR: interquartile range.

In all three groups, most participants were male (G1=51.6%, G2=53.6%, and G3=50.9%), had ischemic stroke (G1=87.9%, G2=84.8%, and G3=95.4%), and did not receive thrombolytic therapy (G1=89.9%, G2=84%, and G3=83.5%). Hypertension (G1=66.1%, G2=67.5%, and G3=59.3%) and sedentary lifestyle (G1=78.5%, G2=78.8%, and G3=80.6%) were the predominant risk factors in all three groups. Mild stroke was more frequent in G1 (50%) and G3 (53.7%), while moderate stroke was the most common in G2 (42.4%) (Table 1). The prevalence of moderately severe or severe functional impairment was higher in G2 (63.6%) than in G1 and G3 (38.8% and 39%, respectively).

**Figure 2:**
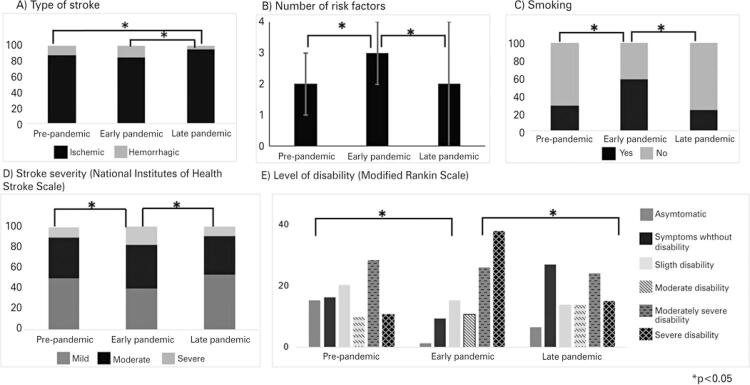
Variables with significant differences between groups (A) Type of stroke; (B) Number of risk factors; (C) Smoking; (D) Stroke severity - National Institutes of Health Stroke Scale; (E) Level of disability - Modified Rankin Scale ^‡^ χ^2;^; ^≠^ANOVA; * p<0.05.

The type of stroke (p=0.02) (Figure 2A), number of risk factors reported (p<0.001) (Figure 2B), risk factor smoking (p<0.01) (Figure 2C), severity of stroke (National Institute of Health Stroke Scale) (p=0.02) (Figure 2D), and level of functional disability (Modified Rankin Scale) (p<0.01) (Figure 2E) was significantly different between the groups.

The number of risk factors (G1 and G2, p=0.02; G2 and G3, p<0.01), smoking (G1 and G2, p<0.01; G2 and G3, p<0.01), severity of stroke (G1 and G2, p=0.03; G2 and G3, p<0.01), and level of functional disability (G1 and G2, p<0.01; G1 and G3, p<0.01) were significantly higher in G2 than in G1 and G3. The type of stroke was significantly different between G1 and G3 (p=0.04) and between G2 and G3 (p<0.01). Moreover, ischemic stroke was most commonly diagnosed during G3 (p=0.02) (Figure 2).

## DISCUSSION

This study compared the sociodemographic and clinico-functional characteristics of patients admitted to a stroke unit of a public hospital from a metropolis in Brazil immediately before and over two different phases of the COVID-19 pandemic. According to the results, individuals affected by stroke at the beginning of the pandemic had greater stroke severity and disability and reported more risk factors, with smoking being the most prevalent, than those affected during the other two phases. Individuals in the late pandemic group had a higher prevalence of ischemic stroke than those in the other groups. Finally, the sociodemographic characteristics of post-stroke patients admitted for stroke did not change owing to the pandemic.

Several high-income countries have reported a reduction in hospitalization of patients with mild stroke, similar to the present study.^(20-22)^ Therefore, there seems to be a global trend in reduced hospital admissions due to acute mild stroke cases after the onset of the pandemic.

Various factors have been suggested to explain the decline in the number of mild stroke cases after the onset of the pandemic.^(2,6,7,23)^ A sudden increase in COVID-19 cases and number of deaths at the beginning of the pandemic led to the abrupt but necessary reorganization of health services that limited the emergency care of acute conditions.^(2,6,7,23,24)^ In addition, social distancing measures advocated to contain the spread of infections in Brazil and worldwide were accompanied by uncertainty.^(9)^ The fear of being infected may have restricted many individuals with mild strokes to their homes, thereby worsening their clinico-functional condition.^(25)^ These factors may justify the reduction in cases of mild stroke in the early pandemic phase but not in the late pandemic phase. After an initial period of abrupt changes in the early phase of the pandemic, the health system may have managed to organize itself to face the pandemic. Additionally, the results suggest that the population’s increasing knowledge of disease transmission and coping measures to deal with COVID-19 may have favored the treatment of acute patients with or without COVID-19.

Consistent with previous studies that reported a delay in the arrival of individuals with stroke at health services immediately after the onset of pandemic, cases with greater severity of stroke associated with more significant functional impairment were observed in group G2 than in the other groups.^(4,6,7)^A possible delay in admission to the health service, screening and establishment of diagnosis, and effective treatment initiation negatively affected the clinico-functional outcome of individuals after a stroke.^(1,4,7,24,26)^Thus, these individuals may require additional rehabilitation services after hospital discharge and in the later chronic phase of stroke.

Individuals with stroke in G2 had more risk factors, with smoking being most prevalent, than those in the other groups. The onset of the pandemic resulted in worsening of pre-existing weaknesses in several health centers responsible for different levels of care.^(27)^ Studies have reported that the role of primary healthcare in the Brazilian public health system has been fundamental in handling the pandemic because this level of care is responsible for managing problems arising from social isolation, such as increase in mental disorders, decrease in physical activity levels, and worsening or appearance of chronic diseases.^(28)^ However, these studies reported a redistribution of health professionals from the primary care sector to the hospital service after the pandemic started, with a possible reduction in prevention and management services directed to the control of the risk factors related to the occurrence of other conditions.^(28)^ In addition, the pandemic and social isolation resulting from confinement can increase unhealthy behaviors, such as a sedentary lifestyle, excessive alcohol consumption, or smoking, and generate negative emotional responses, such as stress and depression.^(29-31)^

Individuals in the G3 group presented with significantly higher proportions of ischemic stroke than those in the other groups. Thromboembolic events, which can occur many days after the disease symptoms, are some of the notable clinical repercussions of COVID-19 and a part of the widely known post-COVID-19 syndrome. It can cause pulmonary embolism and stroke.^(32,33)^As previously reported, COVID-19 patients seem to be more susceptible to developing cerebrovascular diseases, including ischemic stroke, than healthy controls.^(33)^ The number of COVID-19 patients and circulation of the virus in the population was higher in the G3 group than during the early pandemic phase.^(8)^ Thus, the increase in the number of ischemic stroke patients in G3 could be partly be a consequence of COVID-19.^(32,33)^

There were no changes in the sociodemographic characteristics of the post-stroke patients admitted to the stroke unit during the COVID-19 pandemic. In all groups, most of the sample comprised older men from lower socioeconomic classes. These results are consistent with those of studies conducted before the pandemic that investigated the sociodemographic characteristics of post-stroke patients in high-income and middle-income countries.^(34,35)^ Furthermore, these results highlight the practical situation in Brazilian public healthcare services and high demand for care by patients primarily from the lower socioeconomic classes.^(36)^

These study had some limitations. This study did not present any data on the rate of stroke cases with past or active COVID-19 infection. Since COVID-19 infection was demonstrated to cause different forms of ischemic and hemorrhagic vascular complications, this could help explain the prevalence of ischemic stroke in the late pandemic phase. Furthermore, this study included only individuals from a single Brazilian metropolis. Given Brazil’s diversity, further studies must be conducted in different cities and regions.

However, despite these limitations, these results are relevant. The metropolitan region of Belo Horizonte is the third largest urban agglomeration in Brazil, and the hospital where the study was conducted provides assistance to about 1.1 million inhabitants.^(37)^ Therefore, these conclusions may be generalized. Finally, previous studies on the global impact of COVID-19 on stroke did not consider different COVID-19 pandemic phases.^(38,39)^ The present study design improved upon methods employed in previous studies to provide novel and relevant results. First, this study was performed in a middle-income country, where changes in the clinical and functional characteristics of individuals affected by stroke due to COVID-19 remain unknown. Second, this study included all stroke types with all levels of severity. Finally, this study included a prolonged and consecutive COVID-19 pandemic phases, which allowed us to observe possible changes in the sociodemographic and clinico-functional characteristics of individuals from the stroke unit in the different phases.

## CONCLUSION

The present study showed that individuals in the early pandemic phase had a greater number of severe stroke events and higher level of disability. These individuals have a greater need for monitoring rehabilitation services after hospital discharge and in the late chronic phase of stroke. Additionally, individuals in the early pandemic phase had a greater number of risk factors including smoking. Therefore, in periods of health emergencies, such as a pandemic, health promotion and prevention services must be strengthened to prevent other important health problems, such as stroke. Only the occurrence of ischemic stroke increased in the late pandemic period. Thus, the impact of the COVID-19 pandemic on the clinico-functional characteristics of individuals with stroke varied in the different phases. These results should be used for planning health care and managing public policies related to stroke in chronic stages of the disease, even after the pandemic. Future studies should investigate the impact of the pandemic on the different levels of care of individuals with stroke after hospital discharge.
